# Contraceptive use and determinants of unmet need for family planning; a cross sectional survey in the North West Region, Cameroon

**DOI:** 10.1186/s12905-018-0660-7

**Published:** 2018-10-20

**Authors:** Esambe Emmanuel Edietah, Philip Nana Njotang, Atem Bethel Ajong, Marie José Essi, Martin Ndinakie Yakum, Enow Robinson Mbu

**Affiliations:** 1 Department of Obstetrics and Gynaecology, Faculty of Medicine and Biomedical Sciences, University of Yaoundé I, Yaoundé, Cameroon; 2 Obstetrics and Gynaecology unit, Yaoundé Central Hospital, Higher Institute of Health Technologies, Yaoundé, Cameroon; 3 Department of Public Health, Faculty of Medicine and Biomedical Sciences, University of Yaoundé I, Yaoundé, Cameroon; 4 Meilleur Accès aux Soins de Santé, Yaoundé, Cameroon; 5 Directorate of Family Health, Ministry of Public Health, Yaoundé, Cameroon

**Keywords:** Contraceptive use, Determinants, Unmet need, Family planning, Rural Cameroon

## Abstract

**Background:**

Reducing unmet need for family planning by increasing the rate of modern contraceptive use is indispensable if Cameroon must meet maternal mortality targets of the Sustainable Development Goals. The objective of this survey was to estimate the rate of contraceptive use and identify factors associated with unmet need for family planning in rural Cameroon.

**Methods:**

It was conducted a community-based cross sectional survey from February to March 2016 targeting women in a union of the Wum Health District. Participants were included by cluster multistep sampling and data collected by trained surveyors using a pretested questionnaire. Data were analysed using Epi-Info version 3.5.4. The odds ratio was used as a measure of association between unmet need for family planning and selected covariates with the statistical significant threshold set at *p* ≤ 0.05.

**Results:**

Among the 466 women included in the survey, 78.5% were legally married. The mean age of the participants was 28.7 ± 7.2 years with a mean number of years of cohabitation of 9.1 ± 7.4 years. A total of 438 women from the sample were evaluated for contraceptive use and unmet need for family planning. The rate of modern contraceptive use at the time of the survey was 13[10.1–16.6]% and about 5 in every 10 women had an unmet need for family planning (46.6[41.8–51.4]%) with 31.1% having an unmet need for spacing and 15.5% an unmet need for limiting births. The potential demand for contraception was estimated at 45.9% with only 39.8% of this demand met.

When controlled for age, monthly revenue, occupation and partner’s level of education, discussion of family planning within the couple (OR = 0.66[0.44–0.97], *p*-value = 0.032), and partner’s approval of contraception (OR = 0.66[0.45–0.97], *p*-value = 0.035), were found to be significantly associated with decreasing unmet need for family planning.

**Conclusion:**

With the very low rates of modern contraceptive use and potential demand for contraception in the Wum Health District, the rate of unmet need for family planning is still very high. Non discussion of family planning within the couple, and disapproval of contraception by the partner are significantly associated with high unmet need for family planning. More of couple-based family planning interventions should be encouraged.

**Electronic supplementary material:**

The online version of this article (10.1186/s12905-018-0660-7) contains supplementary material, which is available to authorized users.

## Background

Despite the benefits of modern contraception and family planning, especially in protecting the health of women and asserting their reproductive rights, the rate of unmet need for family planning is still very high in the developing world. The prevalence of unmet need for family planning in most of these countries ranges from 15 to 58% [1–5]. Correct use of modern contraceptives remains the best preventive measure to reduce induced non-therapeutic abortions and limit maternal and neonatal morbi-mortality [4, 6]. The rate of voluntary induced abortions in Cameroon remains very high and varies from urban to rural zones. In a cross sectional survey in Cameroon (2011), the rate of voluntary induced abortions in Yaoundé (Urban) was 25.6% against 27.1% in rural setting (Wum District Hospital) [7]. Increasing the use of modern contraception in Cameroon will go a long way to reduce the already high rates of voluntary induced abortions and their complications. Contraceptive methods readily available in Cameroon and more precisely in the North West Region include: implants, intra-uterine device (IUD), combined oral contraceptives (COC), hormonal injectable methods, and condoms. In 2011, the rate of use of these methods among women of child-bearing age in rural Cameroon was as follows: Condoms (3.6%); injectables (2.5%); oral pills (1.3%), implants (0.5%) and IUD (0.2%). IUDs, implants, oral pills and injectables are most often obtained from both private and public health facilities while condoms are easily gotten from shops and road side hawkers [8]. Family Planning services are offered by trained nurses integrated into the prenatal and postnatal packages in some facilities while in some cases, hospitals create special family planning units for the delivery of these services [8].

Findings from the 2011 national Demographic and Health Survey (DHS) in Cameroon state that only 16% of women of childbearing age were using a modern contraceptive method. There was a significant difference between the urban and rural zones (6% rural against 21% urban) and 17% had an unmet need for family planning (22% urban against 24% rural). The rate of modern contraceptive use was 20.9% in the Northwest Region of Cameroon with a 17.7% prevalence of unmet need for family planning. The potential demand for contraception (the proportion of women who had characteristics indicating that they were potentially in need of contraception) in the Northwest Region was at 55.8% with only 68.3% of this demand met as of 2011 (the percentage of women with a potential demand of contraception who were actually using contraception). There was a significant difference between the potential demand for family planning services in urban and rural Cameroon (56.1% urban against 38.7% rural) [8].

In a recent survey in urban Cameroon, about one in every five women of childbearing age had an unmet need for family planning. Partner’s approval of family planning and discussion of family planning within the couples were two factors strongly associated to unmet need for family planning [5]. These factors among others like level of education of the woman, woman’s age, number of living children, woman’s occupation, level of education of the partner have been reported to be associated to unmet need for family planning in the developing world [1, 4, 9].

With the improvement of the family planning delivery services in Cameroon over the years (increased availability of trained personnel, increased awareness of the availability of family planning methods within the population, and the accessibility of these methods at low cost by the population), and the multiple training sessions organised by the government in partnership with foreign non-governmental bodies (with the aim of retraining reproductive health personnel in this domain), the impact of such on unmet need for family planning has not been evaluated in rural Cameroon. The survey carried out in 2015 in urban Cameroon [5] sampled women with socio-economic status and cultural beliefs which are different from those of the Northwest region of Cameroon [8] and the rural population of the Wum Health District in particular. More so, literature on this subject matter in rural Cameroon is practically inexistent.

An estimate of the prevalence of modern contraceptive use, unmet need for family planning in rural Cameroon and its determining factors will help policy makers in resource allocation, modification of existing programmes and the designing of better interventions to increase the uptake of modern contraceptive methods. This will not only significantly reduce the already high rates of maternal morbi-mortality in the developing world but also go a long way to improve family welfare. We therefore designed a survey in a rural Cameroon setting to estimate the prevalence of modern contraceptive use, the unmet need for family planning and identify determinants of unmet need for family planning among women of child bearing age in a union.

## Methods

A community-based cross sectional survey was conducted targeting women of childbearing age (15–49 year) in a union of the Wum Health District (Northwest region of Cameroon) from February to March 2016. The Wum Health District is the largest rural Health District in the region, covering a total surface area of 2700km^2^ with 16 health areas, 02 of which are semi-urban and the rest rural. It covers three of the four administrative Subdivisions that make up the Menchum Division. Eligible participants were women of child bearing age in a union living in the district at the time of the survey.

The minimum sample size for the survey was estimated using the following parameters: The estimated proportion of women in a union with an unmet need for family planning in Cameroon [8], the absolute precision required on either side of the considered proportion (0.05), a cluster effect of 2 and a 95% confidence interval. Using a multistep cluster sampling, 466 eligible and consenting participants were included in the survey. Ten out of the fourteen rural health areas in the Wum Health District were randomly selected with replacement. In each selected health area, all the villages that make up the health area were identified and a village chosen randomly. The chosen villages were divided into quarters and in each village two-thirds of the quarters chosen randomly with replacement. All major road junctions in the selected quarters were identified and at each junction, a street was randomly selected. In each selected street, all households on the left hand side of the surveyor were consecutively included in the survey. In each household, only eligible and consenting participants were included in the survey.

Data were collected using a pretested questionnaire, administered face to face by trained surveyors. To minimize different forms of bias, a two-day training session was organised during which the surveyors were clearly explained the purpose of the research and trained on the consenting and data collection procedure. At the household level, the surveyors introduced themselves and with the help of the head of the house or his representative, eligible participants were identified. Administration of the consent form and the questionnaire (for consenting participants) then followed. Data collected included, but not limited to: sociodemographic characteristics of the population, their knowledge on the different contraceptive methods, adoption of contraception, pregnancy states, past obstetric and surgical history (see questionnaire; Additional file 1).

### Data analysis

The data from all validated questionnaires were double entered into a predesigned data entry sheet, cleaned, compared and analysed using the statistical software Epi-info version 3.5.4. Proportions and their 95% confidence intervals were calculated where necessary for categorical variables and means for continuous variables. The magnitude of unmet for family planning was estimated using the “Westoff/Demographic and Health Survey method” of measuring unmet need, also known as the “core definition method which was adopted in a similar survey in urban Cameroon” [5, 10].

**Fig. 1:**
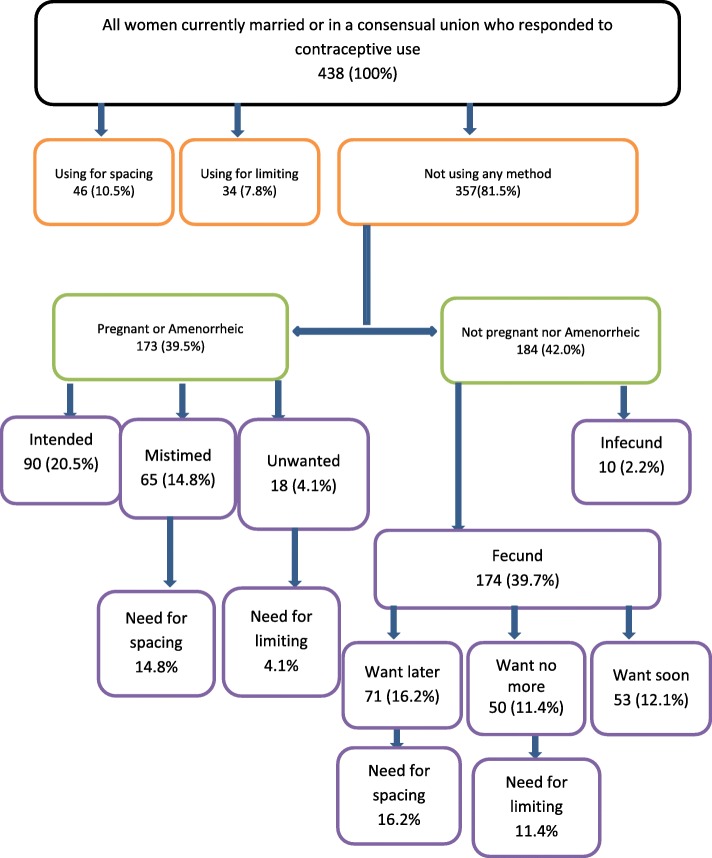
Stepwise evaluation of unmet need for family planning

According to this core definition method, we start by determining the number of women who are in a union but who are currently not using any contraceptive method. This population is further divided into two groups; those who are “pregnant and postpartum amenorrheic” against those who are neither “pregnant nor postpartum amenorrheic”. From the pregnant and amenorrheic group, amenorrheic women who had their last pregnancy planned and pregnant women who declared their pregnancy planned or intended were subtracted leaving us either with those who declared their pregnancies mistimed (wanted more babies but were not prepared to give birth at that time; unmet need for spacing “1”) or unwanted (had stopped child bearing but accidentally got pregnant; unmet need for limiting “1”). From the group of women who were neither pregnant nor amenorrheic, infecund women were excluded and only those that were fertile considered. From the fertile population, those who expressed desires of having more children but at least two years from the day of the survey were considered to have an unmet need for spacing “2”, and those who declared to have stopped childbearing had an unmet need for limiting “2” while fertile women who intended to have babies within the next two years were excluded. Unmet need for spacing was calculated by adding unmet need for spacing “1” to unmet need for spacing “2” while unmet need for limiting was calculated by adding unmet need for limiting “1” to unmet need for limiting “2”. Total unmet need for family planning was calculated as the sum of unmet need for spacing and unmet need for limiting (see Fig. 1).

The potential demand for contraception was then calculated as the sum of the percentage of women currently using contraception and women with unmet need (excluding women with unmet need who were pregnant or postpartum amenorrheic). The current met contraceptive demand of this population was estimated as the proportion of current contraceptive users among those with a potential demand for contraception. Determinants of unmet need for family planning were identified using logistic regression with the odds ratio (and its 95% confidence interval) serving as a measure of association between the outcome of interest (unmet need for family planning) and selected covariates (discussion of family planning among the couple, partner’s approval of family planning, level of education of the participant, and religion). Potential confounders like level of education of the partner, age, and occupation were used in a multiple logistic regression model all with the statistical significant threshold set at *p*-value≤0.05. The confounders were selected based solely on literature.

### Operational terms

- **➢Unmet need for Family planning**: A woman was classified to have an unmet need for family planning in this survey if she was fertile, had no desire for childbearing in her life, or desired to postpone childbearing for at least two years and was not using a contraceptive method. Also, “pregnant or postpartum amenorrheic” women who respectively declared their current pregnancy or their last pregnancy to be unplanned (the pregnancy came as a surprise or when they were not prepared for it, but they however decided to keep the pregnancy) were considered to have an unmet need for family planning. Fertile women who were not using contraception but desired no more children or women who were “pregnant or postpartum amenorrheic” but who respectively declared their current pregnancy or last pregnancy unwanted (to have occurred when they had decided to end childbearing) were classified to have an **unmet need for limiting**. On the other hand, fertile women who were not using contraception but desired postponing childbearing at least for two years or “pregnant or postpartum amenorrheic” women who respectively declared their current or last pregnancy mistimed (occurred at the wrong time but they however had intentions of having more children in their life) were considered to have an **unmet need for spacing**.
- **➢Infertile woman**: A woman who reported to be infertile, or who has undergone hysterectomy, bilateral tubal ligation, or bilateral salpingectomy or has a non-gestational amenorrhoea for more than 1 year or has a postpartum amenorrhoea of more than two years.
- **➢A woman in a consensual union**: A woman living with the partner under the same roof as married without formal ratification by the law for at least six(6) months. Also said to be in a “free union”.
- **➢Woman in a union**: A woman who is either in a consensual union or is married.

## Results

A total of 486 eligible women contacted for the survey. Out of these, 20 did not consent to the survey (non-response rate of 4.1%). Among the 466 eligible and consenting participants, 78.5% were married and the rest in a consensual union. The mean age of the participants was 28.7 ± 7.2 years with a mean number of years of cohabitation of 9.1 ± 7.4 years. About 9 in every 10 women surveyed were Christians; 6 of which were Catholics. More than half (58.2%) of the women surveyed had acquired just primary education while 34.8% attended at least secondary school. Most (97.0%) of the population declared having an estimated monthly revenue of less than 50000FCFA (80.75 US dollars).

About 9 in every 10 women surveyed [438(94.0%)] had heard of contraception with 426(91.4%) able to state the use of contraception. The most commonly cited contraceptive methods were the male condom (91.6%), injectables (62.0%) and oral pills (59.4%). Among the 438 women who responded to contraceptive use, 18.3[14.8–22.3]% were currently using a contraceptive method with a modern contraceptive use rate of 13.0[10.1–16.6]%. The rate of use of the male condom was very low (4.7%) and only 1.5% of the sample adopted long acting reversible contraceptive methods (IUD and implant).

Figure 1 shows a step wise estimation of the unmet need for family planning in the sampled population. The total unmet need for family planning among women in a union was estimated at 46.6[41.8–51.4] % with 31.1% having an unmet need for spacing and 15.5% an unmet need for limiting. The rate of unplanned pregnancy (proportion of women who reported at least one unplanned pregnancy in their life) among women with unmet need (50.5%) was more than doubled the rate of unplanned pregnancy among women with met need for family planning (20.4%). The potential demand for contraception [the percentage of current contraceptive users added to that of women withunmet need for family planning (excluding women with unmet need who were pregnant or amenorrheic)] was estimated at 45.9% (10.5% + 7.8% + 16.2% + 11.4%). The potential of contraception for spacing was estimated at 26.7% (10.5 + 16.2%) and the demand for limiting at 19.2% (7.8% + 11.4%). Only about 4 in every 10 women (39.8%) with a potential contraceptive demand were currently using a modern contraceptive method.

Table 1 shows the determinants of unmet need for family planning evaluated with simple and multiple logistic regressions; with the odds ratio as a measure of the strength of association. Level of education above secondary, partner’s approval of contraception, and discussion of family planning within the couple were significantly associated with decreasing unmet need for family planning following univariable analysis..

**Table 1:** Determinants of unmet need for family planning

Factor	Univariate analysis			Multivariate analysis		
	OR	CI 95%	*p*-value	Adj OR	Adj CI 95%	*p*-value
Level of education above secondary (Y/N)	0.45	0.21–0.97	0.041*	0.61	0.26–1.45	0.264
Partner’s approval of contraception (Y/N)	0.64	0.44–0.94	0.023*	0.66	0.45–0.97	0.035*
Discussion of family planning within the couple (Y/N)	0.64	0.44–0.94	0.023*	0.66	0.44–0.97	0.032*
Religion Catholic (Y/N)	0.9	0.62–1.32	0.601	0.90	0.61–1.32	0.592
Number of children alive > 5 (Y/N)	0.81	0.49–1.36	0.425	0.80	0.47–1.37	0.426

*Y/N* = Yes/No, *OR* = Odds Ratio, *CI*=Confidence Interval, *Adj OR* = Adjusted Odds Ratio and *Adj CI* = Adjusted Confidence Interval *statistically significant (*p*-value≤0.05)

When controlled for age, monthly revenue, occupation and partner’s level of education in a multiple logistic regression model, discussion of family planning within the couple (OR = 0.66[0.44–0.97], *p*-value = 0.032), and partner’s approval of contraception (OR = 0.66[0.45–0.97], *p*-value = 0.035), were found to be significantly association with decreasing unmet need for family planning.

## Discussion

Among the 466 women who were included in the survey, 78.5% were legally married. The mean age of the participants was 28.7 ± 7.2 years with a mean number of years of cohabitation of 9.1 ± 7.4 years. A total of 438 women from the sample were evaluated for contraceptive use and unmet need for family planning. The rate of modern contraceptive use at the time of the survey was 13[10.1–16.6]% and about 5 in every 10 women had an unmet need for family planning (46.6[41.8–51.4]%) with 31.1% having an unmet need for spacing and 15.5% an unmet need for limiting births. The potential demand for contraception was estimated at 45.9% with only 39.8% of this demand met. When controlled for age, monthly revenue, occupation and partner’s level of education, discussion of family planning within the couple (OR = 0.66[0.44–0.97], *p*-value = 0.032), and partner’s approval of contraception (OR = 0.66[0.45–0.97], *p*-value = 0.035), were found to be significantly associated with decreasing unmet need for family planning.

The rate of modern contraceptive use reported in this survey is relatively low compared to national statistics in urban Cameroon but however about doubled the rural modern contraceptive prevalence reported in the 2011 demographic and health survey (6%) [8]. Even though the counselling and sensitization on contraception in recent years is more focused on the adoption of long acting reversible contraceptive methods (LARCs), the rate of use of LARCs (implants and IUDs) within the sampled population was still very low. The seemingly increasing rate of contraceptive use compared to data from rural Cameroon in 2011 could be explained by the different efforts put in by the Cameroon government in collaboration with external non-governmental bodies to educate the potential clients on contraception while improving accessibility to different contraceptive methods.

The relatively low rate of modern contraceptive use in this population compared to the urban Cameroon settings could be explained [8]. Rural areas are characterised by poor geographical accessibility, little or no audio-visual signal coverage and therefore compromised access to information as far as contraceptive practices are concerned; About half and more of the sampled population had attended only primary school and might not be able to understand the official languages used on TV and radio. Moreover, the cultural beliefs and conservatism of the population sampled could make persuasion by the family planning care-givers difficult. In addition, the misconceptions associated to different modern contraceptive methods could affect their perceptions and therefore attitude towards these methods of birth control [11–14]. These and others might explain the discrepancies between the results in our setting and urban settings. Our findings however concord with the low modern contraceptive rates reported during similar a cross sectional survey among women in rural Burkina Faso in 2014 [15].

The low rate of modern contraceptive practice recorded in this survey could have serious reproductive health implications. Low rates of modern contraceptive practice have been associated with increasing rates of unintended pregnancy, school dropouts, induced abortions and maternal morbi-mortality. This is a call to policy makers to allocate more resources or to redesign interventions towards scaling up the use of modern contraception in rural Cameroon.

The level of unmet need for family planning reported in this survey is relatively very high. According to our findings, about one in every two women in the sampled population had an unmet need for family planning with the unmet need for spacing about doubled the unmet for limiting. This high level of unmet need for family planning is expected in a population with only 13% rate of modern contraceptive use. Our results are in line with the findings on unmet need for contraception in Central Ethiopia [2] and rural Burkina Faso [15] but comparatively higher than results obtained in the 2011 DHS in Cameroon [8], in urban Cameroon in 2015 [5], Nigeria in 2013 [16], Eastern Sudan [4] and in Dangila [3]. This high rate of unmet need in the sampled population can be explained by similar reasons associated to low contraceptive uptake given above. The low modern contraceptive prevalence and very high unmet need for family planning could explain the low potential demand for contraception. As already presented above, the rate of unplanned pregnancy among women with unmet need for family planning was more than doubled that in women with met contraceptive needs. In other words, women with an unmet need for family planning were twice as likely to have had an unplanned pregnancy during their reproductive life compared to women without met contraceptive needs. A high rate of unplanned pregnancy is usually associated to a high rate of voluntary induced abortions. Given that induced non-therapeutic abortions are not legalised in Cameroon, abortion seekers tend to frequent unqualified care-givers or resort to delicate and life-threatening practices with the aim of destroying or sending out the product of conception. These practices are usually associated to high rate of post-abortion complications and contribute to keep maternal mortality high. This high rate of unplanned pregnancy among women with unmet need for contraception could explain the high rates of voluntary induced abortions reported by Ngowa et al. in the Wum Health District [7]. The low rate of met contraceptive demand reported in this survey is a call to the government and family planning providers to improve access to contraception and modify the sensitization and persuasion techniques in order to break the cultural barriers and beliefs associated with family planning.

Women who discussed about family planning with the male partners were 0.66 times less likely to have an unmet need for family planning than those who did not. Approval of family planning and contraception by the male counterpart was also associated to a significant decrease in the likelihood of unmet need for family planning. A recent survey carried out in urban Cameroon pointed out these two factors to have high statistical significant associations with unmet need for family planning [5]. Several African surveys have also identified the impact of the male partner in contraceptive decision making and uptake by the woman [2, 3, 15].

These two factors highlight the part played by the male counterpart in contraceptive decision making. Our results in a rural Cameroon setting coupled to the findings in urban Cameroon and other parts of the world reveal non-discussion of family planning within the couple and partner’s disapproval of contraception as two significant barriers to the effective adoption of modern contraception. Even though some women in a union still adopt modern contraceptive methods without the consent of their partners, the push or pull towards adoption of modern contraception depends on the attitude of the male counterpart towards these reproductive health services. Family planning programmes in Cameroon have made significant progress towards promoting the use of modern contraception by concentrating efforts on the woman but “couple-based counselling” seems more than indispensable if the sustainable development goal target on maternal and neonatal morbi-mortality is to be met in Cameroon.

This strong influence of the male counterpart could be associated to the nature of the African society, where issues of gender-based violence and female emancipation are still confronted with strong rejection. Programmes with goals of preventing reproductive coercion in our context are practically inexistent. Decisions on the number of children and when to have these children are generally imposed by the man on the woman probably because of reduced financial autonomy among women. Family planning interventions in Cameroon should target both men and women, and counselling towards contraceptive uptake be made more of a “couple thing”. Also, efforts towards limiting gender-based violence and promoting female emancipation while educating the population for a change in attitude towards modern contraceptive methods could also allow the woman with some degree of autonomy in decision making and increase contraceptive use in our context.

### Limits and strengths of the survey

The results of this survey should be interpreted with care. The cross sectional design of this survey does not permit a direct establishment of cause-effect relationships. Our survey did not evaluate the unmet need for family planning among women out of union, among men and the couples’ unmet need. This is important given that our findings pointed to a strong influence of the male partner in contraceptive decision making. In addition, the competence of the family planning providers and the supply of contraceptive methods in this population were not evaluated in our survey. However, the results obtained following norms of scientific research and with adequate methodology are in absolute conformity with previous research findings. Our study serves as an indicator to family planning needs and will help in the adequate allocation of family planning resources in Cameroon.

## Conclusion

The rate of modern contraceptive use and the potential demand for contraception in the Wum Health District is still very low. About half of the women in a union in the Wum Health District have an unmet need for family planning. Factors significantly associated to high unmet need for family planning in Cameroon are non-discussion of family planning within couples, and disapproval of family planning by the male partner. Family planning interventions should thus be more focused on the couple and not just the woman. Our results were obtained only from women but given the findings of the survey indicating the vital role of the male counterpart in decision making, research in this domain should move on to evaluate the unmet need for family planning among men and the couples’ unmet need in Cameroon. Also, other studies should be carried out to identify barriers to autonomous decision making as far as contraception is concerned among women. The competence of the family planning providers and its supply in Cameroon also need evaluation.

## Additional file

Additional file 1: Questionnaire for the evaluation of contraceptive use, unmet need for family planning and its determinants. (DOCX 146 kb)

## Abbreviations

AOR: Adjusted Odds Ratio
CI: Confidence Interval
COC: Combined Oral Contraceptives
DHS: Demographic and Health Survey
IUD: Intra-Uterine Device
LARCs: Long Acting Reversible Contraceptives
