# Supplementary material for: Contraceptive use and determinants of unmet need for family planning; a cross sectional survey in the North West Region, Cameroon
Source: BMC Womens Health. 2018 Oct 20;18:171. doi: 10.1186/s12905-018-0660-7 (PMC6195995; doi:10.1186/s12905-018-0660-7)
Supplement: Supplementary file 1 — Questionnaire for the evaluation of contraceptive use, unmet need for family planning and its determinants. (DOCX 146 kb) [file 12905_2018_660_MOESM1_ESM.docx]

**Questionnaire**

Date………………………………………

Name of surveyor……………………………………………..

Code of participant: …/…/…/…/…..

**General characteristic, Socio-demographic and economic data**

1. Age: …………………………………………………………………………..
2. Health area/Quarter …………………………………………………………………….
3. Marital status: Married single Divorced free union
4. Religion: Catholic Protestant Muslim Atheist Animist

Pentecostal

1. Number of years of marriage/ cohabitation……………………
2. Level of education: Never schooled Primary secondary higher
3. Educational level of the partner: Never schooled Primary secondary

Higher

1. Occupation: Skilled job unskilled Student Nothing
2. What’s your estimated monthly revenue? Less than 50000, 50000-100000,

100000-200000 200000-250000 above 250000

1. Total number of pregnancies………… number of pregnancies planned for ……………… Number of children alive ………………………… Age of last child ………………………
2. How many children do you intend to have in your life? ……………………………………….
3. LMP: …………………………………. If none, for how long have you not menstruated? ..............................................................................................................
4. Any history of surgery? Precise …………………………………… Have you been diagnosed with any infertility issues? ……………………, if yes, precise ……………………………………..

**Knowledge**

1. Have you ever heard of contraception? Yes No
2. When do you think we have need for contraception? Limitation of birth

prevention of unwanted pregnancy help prevent STI when am pregnant

To space births no idea.

Others…………………………………………………………………………………

1. Who should use contraception? Married women unmarried women all sexually active women. Only women with at least one child

Others…………………………………………………………………………………

1. Where do you think we can get modern contraceptives? in the hospital

Hawkers at the pharmacy in shops,

others……………………………………………………………………………………

1. Can you cite the different contraceptive methods you know?

M. Condoms F. condoms diaphragms cervical caps IUD

spermicides Implants COC injectables LAM coitus interuptus Others…………………………………………………………………………………Which of the above methods prevent STI/HIV transmission?

M. Condoms F. condoms diaphragms cervical caps IUD

spermicides Implants COC injectables LAM coitus interuptus Others…………………………………………………………………………………What is your main source of information concerning contraception?

Radio/ TV, health personnel family and relations at school

v

1. According to you, what is the best source of information about contraception?

The media Health personnel family and relations, friends, others………………………………………….

**Evaluation of unmet contraceptive needs and contraceptive preference**

1. Do your beliefs approve of contraception? Yes No
2. If no, why? It changes the natural pattern of life, my religion does not allow it It promotes sexual promiscuity. Others…………………………………………………………………………………
3. Does your partner approve of contraception? Yes No
4. How often do you discuss about family planning with your partner? Never

Once or twice more often

1. Do you have any intentions of giving birth again? Yes No. If yes, how long from now? ………………….
2. Have you ever used any of the above cited contraceptive methods? Yes No
3. If yes, which of them?

M. Condoms F. condoms diaphragms cervical caps IUD

spermicides Implants COC injectables LAM coitus interuptus Others…………………………………………………………………………………

1. From your first sexual contact, what is the main contraceptive method you have used?

M. Condoms F. condoms diaphragms cervical caps IUD

spermicides Implants COC injectables LAM coitus interuptus Others…………………………………………………………………………………

1. Are you presently using a contraceptive method? Yes No.
2. If yes, which one(s)

M. Condoms F. condoms diaphragms cervical caps IUD

spermicides Implants COC injectables LAM coitus interuptus Others…………………………………………………………………………………

1. If you are currently not using a method, have you plans of using contraception in the future Yes No
2. Are you presently pregnant? Yes No

C

C

1. If yes, is this pregnancy desired or timed? Yes No

C

C

1. Have you ever been pregnant? Yes No

C

C

1. If yes, was your last pregnancy desired and or timed? Yes No

C

C
